# Pseudobulbar laughter as a levodopa off phenomenon exacerbated by subthalamic deep brain stimulation

**DOI:** 10.1186/s40734-015-0023-6

**Published:** 2015-08-14

**Authors:** P. K. Chattha, P. E. Greene, Ritesh A. Ramdhani

**Affiliations:** Medical University of Lublin, Lublin, Poland; Department of Neurology Division of Movement Disorders, Icahn School of Medicine at Mount Sinai, New York, NY 10029 USA; Department of Neurosurgery, Icahn School of Medicine at Mount Sinai, 5 East 98th St. First Floor, New York, NY 10029 USA

**Keywords:** Pseudobulbar affect, Off phenomenon, Deep brain stimulation

## Abstract

Pseudobulbar affect is a common symptom in neurodegenerative diseases and can also result from lesions in cortical, subcortical and brainstem regions. In Parkinson’s disease (PD), pseudobulbar affect (PBA) can occur as a wearing off phenomenon, manifested usually as crying without emotionality. In addition, subthalamic (STN) deep brain stimulation (DBS) has been reported to induce PBA in PD patients with no prior history of such episodes.

We present a case of inappropriate laughter lacking mirth as a levodopa OFF phenomenon in a patient with PD, whose laughter also worsened with STN-DBS in his non-medicated state. Levodopa ameliorated his PBA both with and without stimulation.

The case demonstrates pseudobulbar laughter as a levodopa OFF phenomenon that is also exacerbated by STN-DBS.

**Electronic supplementary material:**

The online version of this article (doi:10.1186/s40734-015-0023-6) contains supplementary material, which is available to authorized users.

## Background

Pseudobulbar affect (PBA) is described as uncontrollable and inappropriate laughter or crying. It occurs without environmental or internal cues and can be conflicting with an individual’s emotional intentions. It is commonly associated with neurodegenerative disease (i.e., ALS, PSP) as well as with lesions [1–4] in the cortex, subcortical white matter, and brainstem, oftentimes resulting in dysfunction of the corticobulbar tract. The prevalence of PBA in patients with Parkinson’s disease is 5–17 % [5], and has been reported as a stimulation induced symptom in patients with subthalamic (STN) deep brain stimulation (DBS) [6–8]

We report a case of pseudobulbar laughter in a patient with PD as a levodopa off phenomenon that is further exacerbated by STN-DBS.

## Case report

A 52-year-old man with Parkinson’s Disease with STN-DBS presented with pseudobulbar laughter in the OFF medication/ON Stimulation state. At the age of 40, the patient developed tremors in his fingers accompanied by stiffness in his limbs and asymmetric left side bradykinesia. In the ensuing years, he was plagued by motor fluctuations and dyskinesia with unpredictable offs and freezing of gait. His presurgical neuropsychiatric assessment did not reveal underlying depression or anxiety. He was never treated with antidepressants or antipsychotic medications. On the morning of his implantation of bilateral subthalamic nucleus (STN) DBS at an outside institution, he reports that he had bouts of uncontrollable laughter without mirth for the first time in the surgical waiting area prior to be taken into the operating room. He was supposedly off of his PD medications for at least 12 h at that time. Following the surgery, which occurred without complications, attempts by other clinicians to program his DBS while he was off his PD medications apparently produced inappropriate episodes of laughter. Over the next 9–12 months, though his dyskinesia improved, he continued to have motor fluctuations with no evidence of PBA.

**Fig. 1:**
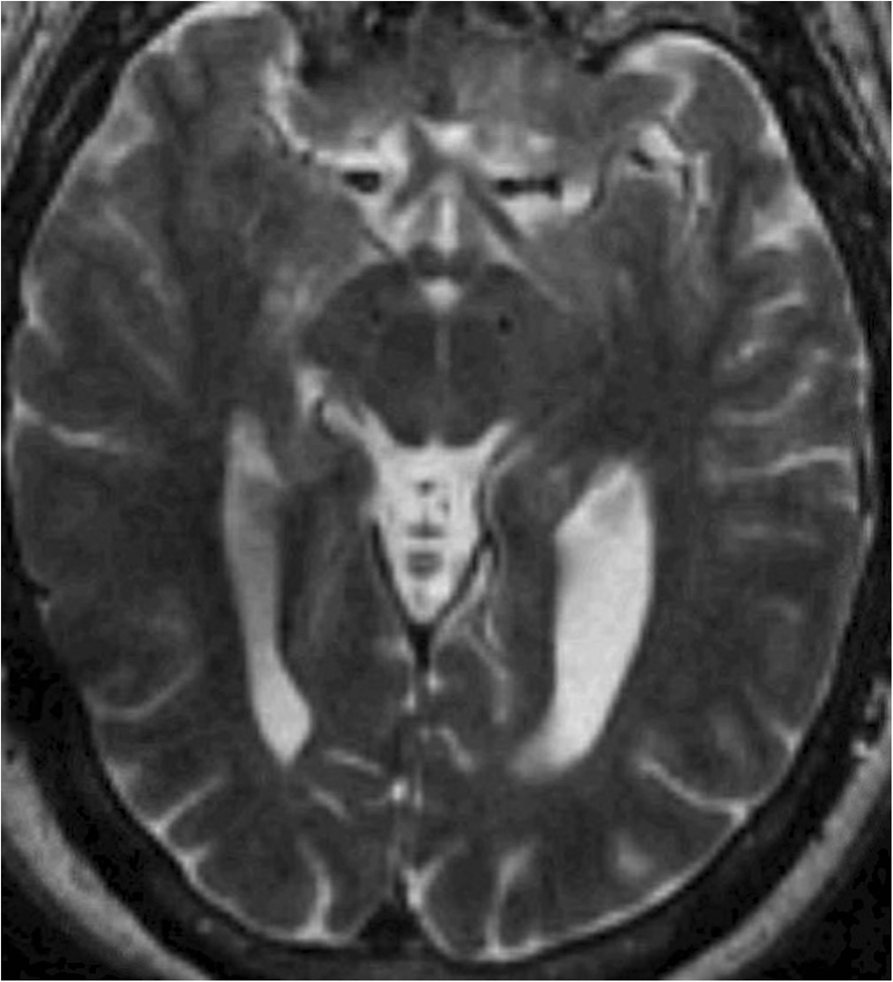
Axial T2 magnetic resonance image showing DBS lead tips in the subthalamus region, approximately 9 mm from the midline on both sides

In order to optimize his programming, he was brought in off all antiparkinsonian medications for 12 h. At the time of this evaluation, his affect was gleeful with periods of non-mirthful chuckling. After his DBS was inactivated for 45 min, he began having inappropriate bouts of laughter that would occur when doing simple speech tasks (i.e., reciting months of the year). A monopolar review was conducted in the OFF medication state. Monopolar stimulation at all contacts (2 V, PW 60 μs Frequency 130Hz) increased the frequency of laughter with a greater effect produced from the ventral contacts (0 & 1). There was no hemispheric preference for this stimulation effect and higher voltages (4 V) at the ventral contacts exacerbated severity of laughter while concomitantly producing facial flushing and diaphoresis. Cessation of stimulation reduced severity of his laughter as well as autonomic facial changes immediately. Sham stimulation failed to reproduce inappropriate laughter. In the ON Medication/ OFF stimulation state, the patient’s UPDRS III score was 13, while in the OFF Medication/ON stimulation it was 26.5. This difference was a result of his axial and gait scores being more responsive to levodopa. Review of his post-operative brain MRI did not show that his leads were malpositioned (Fig. 1) as the centroid of both lead tips were approximately 9 mm from the midline. The monopolar review did not reveal muscle contractions, diplopia, dysarthria, or ataxia—providing functional support that the leads were in the subthalamus region. Furthermore, in the medicated state, regardless of whether he was receiving stimulation or not, his affect was appropriate with no signs of inappropriate laughter. (Refer to Additional file 1: Video 1)

## Discussion

This case describes pseudobulbar laughter as a levodopa off phenomenon. Non-motor symptoms (NMS) of Parkinson’s disease include anxiety, mood changes, and panic attacks, as well as autonomic changes [9] (e.g., sweating, abdominal discomfort, numbness, and pain) Such symptoms can occur as an OFF phenomenon that improves with dopaminergic therapy [9]. Pseudobulbar affect is not uncommon in PD especially in the advanced stages. Inappropriate crying is the most common manifestation of PBA in this disease [10] though it is often associated with a state of depression. It has also been described as occurring in the Off medication state [6]. Our patient’s bouts of laughter, which lacked mirth, is not only indicative of PBA, but its onset and resolution as a function of levodopa dosing supports it as an non-motor OFF phenomenon. Lack of levodopa tends to produce a dysphoric state, which makes the presence of non-mirthful laugher in such a condition very interesting and difficult to differentiate as a product of an emotional stimulus despite this patient not having a history of depression or being on antidepressants.

Pseudobulbar affect has also been reported as a stimulation induced effect in PD patients with STN-DBS (see Table 1). Krack et al. [7] demonstrated acute mirthful laughter in two PD patients with STN DBS, stimulated at either a ventral or dorsal contact. Stimulation activation patterns also seem to have little bearing on inducing PBA as both monopolar [6, 7] and bipolar [8] stimulation have been implicated. The possibility of carryover effect or incomplete washout of stimulation is significantly reduced given the prolonged period of time (45 min) that our patient was off stimulation [11]. The only other reported case of pathological laughter related to stereotactic surgery occurred in a patient post thalamotomy [12].

**Table 1:** Stimulation and Stereotactic Surgical Causes of PBA

Study	n	Age (years)	Anatomical location	Stimulation parameters	On/OFF levodopa	Pseudobulbar affect
Okun et al. [6]	1	46	Left subthalamic nucleus	Monopolar stimulation:	OFF	Crying
				1.5 V, 90 μs, 130Hz		
Krack et al. [7]	2	47	Bilateral Subthalamic nucleus	Monopolar Stimulation:	NA	Mirthful Laughter
				3.6 V/90 μs/160Hz		
		54	Right Subthalamic nucleus	Monopolar Stimulation:	NA	Mirthful Laughter
				5.0–5.5 V/60 μs/130Hz		
Low et al^.^ [8]	1	48	Bilateral subthalamic nucleus	Monopolar stimulation:	OFF	Crying
				0.5 V/60 μs/130Hz		
				Bipolar stimulation:		
				>1.0 V/60 μs/130Hz		
Okun et al^.^ [11]	1	46	Right ventral intermedius nucleus (VIM)	Thalamotomy	NA	Laughter

*NA*, not available

The acute worsening of non-mirthful laughter was evident with increased stimulation in our patient through all contacts, though there was a clear propensity for greater effect among the ventral contacts. The STN has several functional regions including: 1) the dorsolateral area, which governs motor control; 2) the ventromedial region that interfaces with limbic circuits. The medial STN is also adjacent to the lateral hypothalamus and embryologically develops along with it. It is plausible that medial STN stimulation impacts cognitive and emotional networks thus triggering PBA. Furthermore, the facial flushing and diaphoresis observed at higher voltages through the ventral contacts, not only supports medial spread of stimulation but also effects on the lateral hypothalamus which could also potentiate it as a limbic release phenomenon.

The fasciorespiratory center lies in the brainstem and was postulated by Wilson [13] that unknown descending cortical pathways, which when disconnected by a lesion, releases voluntary control of the involuntary laughter and crying centers. Cortical, subcortical and brainstem lesions [14] have been associated with PBA, with a quantitative MRI study among MS patients with PBA [15] revealing distribution of putative lesions in the prefrontal, parietal and brainstem. Cognitive studies [16, 17] suggest that the prefrontal cortex is a mediator of emotional expression that receives input from the parietal cortex and also sends projections to the hypothalamus and brainstem. In addition, the cerebellar outflow pathways may also serve as a modulator of involuntary expression of emotion [18]. Therefore, stimulation in fiber tracts surrounding the STN (caudal zona incerta, medial forebrain bundle, prelemniscal, and caudal internal capsule [8]) could possibly inhibit the voluntary brainstem emotional expression circuit, thus resulting in pseudobulbar laughter. The fact that stimulation worsened PBA in our patient further suggests that the emotional expressivity network may have been primed in the OFF state.

## Conclusion

This case highlights pseudobulbar laughter as a novel Parkinson’s off phenomenon that is negatively affected by STN-DBS, yet ameliorated, like all other non-motor offs, with levodopa.

## Consent statement

The patient has provided consent for this report and the video that accompanies it.

## Additional file

Additional file 1: Video 1. Inappropriate laughter OFF levodopa, that is worsened by increases in stimulation (2 V and 4 V), which subsequently improves in the medicated state. (MP4 20735 kb)

## References

[1] Kim JS (1997). Pathological laughter and crying in unilateral stroke. Stroke..

[2] Martin JP (1950). Fits of laughter (sham mirth) in organic cerebral disease. Brain..

[3] Allman P (1994). Poststroke pathological laughing and crying. Am J Psychiatry.

[4] Virani MJ, Jain S (2001). Trigeminal schwannoma associated with pathological laughter and crying. Neurol India..

[5] Miller A, Pratt H, Schiffer RB (2011). Pseudobulbar affect: the spectrum of clinical presentations, etiologies and treatments. Expert Rev Neurother..

[6] Okun MS, Raju DV, Walter BL, Juncos JL, DeLong MR, Heilman K (2004). Pseudobulbar crying induced by stimulation in the region of the subthalamic nucleus. J Neurol Neurosurg Psychiatry..

[7] Krack P, Kumar R, Ardouin C, Dowsey PL, McVicker JM, Benabid AL (2001). Mirthful laughter induced by subthalamic nucleus stimulation. Mov Disord..

[8] Low HL, Sayer FT, Honey CR (2008). Pathological crying caused by high-frequency stimulation in the region of the caudal internal capsule. Arch Neurol..

[9] Seki M, Takahashi K, Uematsu D, Mihara B, Morita Y, Isozumi K (2013). Clinical features and varieties of non-motor fluctuations in Parkinson’s disease: a Japanese multicenter study. Parkinsonism Relat Disord..

[10] Strowd RE, Cartwright MS, Okun MS, Haq I, Siddiqui MS (2010). Pseudobulbar affect: prevalence and quality of life impact in movement disorders. J Neurol..

[11] Cooper SE, McIntyre CC, Fernandez HH, Vitek JL (2013). Association of deep brain stimulation washout effects with Parkinson disease duration. JAMA Neurol..

[12] Okun MS, Heilman KM, Vitek JL (2002). Treatment of pseudobulbar laughter after gamma knife thalamotomy. Mov Disord..

[13] Wilson SA (1924). Original papers: some problems in neurology. J Neurol Psychopathol..

[14] Siddiqui MS, Fernandez HH, Garvan CW, Kirsch-Darrow L, Bowers D, Rodriguez RL (2009). Inappropriate crying and laughing in Parkinson disease and movement disorders. World J Biol Psychiatry..

[15] Ghaffar O, Chamelian L, Feinstein A (2008). Neuroanatomy of pseudobulbar affect : a quantitative MRI study in multiple sclerosis. J Neurol..

[16] Feinstein A, O’Connor P, Gray T, Feinstein K (1999). Pathological laughing and crying in multiple sclerosis: a preliminary report suggesting a role for the prefrontal cortex. Mult Scler..

[17] McCullagh S, Moore M, Gawel M, Feinstein A (1999). Pathological laughing and crying in amyotrophic lateral sclerosis: an association with prefrontal cognitive dysfunction. J Neurol Sci..

[18] Parvizi J, Anderson SW, Martin CO, Damasio H, Damasio AR (2001). Pathological laughter and crying: a link to the cerebellum. Brain..

